# Predicting elevated transcranial doppler velocity among patients with sickle cell anemia in Uganda: A cross-sectional study

**DOI:** 10.1371/journal.pone.0351700

**Published:** 2026-06-23

**Authors:** Doreen Nayiga, David Mukunya, Simon Odoch, Faith Oguttu, Ian Munabi, Milton W. Musaba, Charles Kimbugwe, Brian Tonny Makoko, Jonathan Babuya, Joshua Mugabi, Alain Nyalihama, Martin Chebet, Lisa Rynn, Samuel Kizito, Vincent Ssentumbwe, Sarah Kiguli, Peter Olupot Olupot

**Affiliations:** 1 Department of Pediatrics and Child Health, Busitema University, Mbale, Uganda; 2 Department of Community and Public Health, Busitema University, Mbale, Uganda; 3 Department of Anatomy, Makerere University, Kampala, Uganda; 4 Department of Obstetrics and Gynecology, Busitema University, Mbale, Uganda; 5 Mbale regional referral hospital, Mbale, Uganda; 6 Silver School of Social Work, New York University, New York, New York, United States of America; 7 Division of Computational and Data Science, Washington University, St. Louis, Missouri, United States of America; 8 Department of Pediatrics and Child Health, Makerere University, Kampala, Uganda; University of Illinois at Chicago, UNITED STATES OF AMERICA

## Abstract

**Background:**

Sickle cell anemia (SCA) is an autosomal recessive blood disorder resulting from a specific point mutation in the β-globin gene. Over half a million children are born with sickle cell anemia annually. Transcranial Doppler (TCD) velocity is an accurate predictor of the risk of stroke among children with sickle cell anemia. Unfortunately, TCD screening is not routinely done in developing countries due to limited resources. There is a need to develop a model that predicts elevated TCD velocity, utilizing routinely collected data to guide management of children with sickle cell anemia.

**Methods:**

We conducted a cross-sectional study from 1^st^ July 2024–30^th^ August 2024 among children with SCA attending the Sickle Cell Clinic. We developed a risk-prediction model for elevated TCD (≥ 170 cm/s) using sociodemographic, hematological, and clinical factors.

We used the least absolute shrinkage and selection operator (LASSO) penalized regression to select the best subset of predictors of increased TCD velocity. Model performance was assessed by determining the discrimination using the area under the curve and calibration by drawing a calibration plot.

**Results:**

We enrolled 385 children; the mean age was 10.3 (SD 3.8) years. The prevalence of elevated TCD, defined as ≥170 cm/s was 8.3% (95% CI: 5.8, 11.5; n = 32/385). Using a lambda of 0.008, the final model had 12 predictors. The predictors included neuropathy, red blood cell count, heart rate, age, adherence to hydroxyurea, headache, hematocrit, serum lactate dehydrogenase, gender, malnutrition, blood transfusion, and neutrophils. The model predicted elevated TCD with an AUC of 84.7% (95%CI: 74.7, 90.8).

**Conclusion:**

We developed and validated a model to predict elevated TCD among children living with SCA in Uganda. Further exploration is needed to assess whether this model predicts stroke.

## Introduction

Sickle cell anemia (SCA) is an autosomal recessive blood disorder resulting from a specific point mutation in the β-globin gene, which results in substitution of glutamic acid to valine at position 6 of the β-globin chain [1]. Globally, more than half a million children are born with sickle cell anemia annually [2], and 80% of these are born in sub-Saharan Africa [3]. Uganda is ranked 5^th^ among countries with the highest incidence of sickle cell anemia [4]. Children with sickle cell anemia (SCA) have a 30% life time risk of developing stroke [5,6]. This is 200-fold higher than the general pediatric population [5]. Children with sickle cell anemia in developing countries are more likely to develop complications such as stroke compared to those in developed countries [7]. This is due to limited access to TCD velocity screening services [8], drugs such as hydroxyurea, folic acid, penicillin, as well as blood transfusions [9,10]. In Uganda, the prevalence of stroke among children with SCA is 6.8% [11].

TCD velocity predicts risk of stroke among children with sickle cell anemia [12]. Children with Timed Average Mean Maximum Velocity (TAAMMV) greater than or equal to 2000 cm/s are classified as high risk. TCD screening followed by prompt management of high-risk patients has been shown to reduce the incidence of stroke among children with SCA by 70% [9,13]. The American Society of Hematology guidelines recommend annual TCD screening for children with sickle cell anemia from age 2–16 years [14]. Unfortunately, TCD screening is not routinely done in developing countries due to limited ultrasound machines, as well as a lack of qualified practitioners to perform TCD screening.

There is a need to generate data to guide accurate prediction of elevated TCD among children with SCA. This will guide screening for children at risk of developing stroke. A prediction index will also guide utilization of limited available resources, such as hydroxyurea and blood transfusion, for the patients considered to be at higher risk. Using data collected from a cross-sectional study among children with sickle cell, we utilized prediction modelling techniques to: [1] identify a model comprising a combination of a subset of multi-level factors that best predicts individualized risk of elevated TCD velocity among children with SCA. [2] Evaluate the extent to which the derived model can discriminate between normal and elevated TCD velocity among children with SCA. [3] Assess the agreement between model-predicted and actual elevated TCD velocities among children with SCA (calibration).

## Methods

### Study design, setting, and participants

We conducted a cross-sectional study from 1^st^ July 2024–30^th^ August 2024 among children with SCA attending the Sickle Cell Clinic at Mbale Regional Referral Hospital (MRRH), a tertiary hospital serving a population of about 4.6 million people in Eastern Uganda. The Sickle Cell Clinic has over 800 clients enrolled in it. The clinic runs every Wednesday and is facilitated by 7 pediatricians, 10 senior house officers, one medical officer, 8 intern doctors, 4 nurses, and 3 clinical officers. The clinic provides hydroxyurea treatment to the patients when in stock at dose ranges of 15–20 mg/kg.

We included children aged 2–16 years with SCA attending the Sickle Cell Clinic at MRRH who were clinically stable. We excluded patients with current presentation of an acute illness such as pain crisis, fever, acute chest syndrome, or other SCA-related acute complications; these were admitted and given appropriate treatment. Patients who had received a blood transfusion in the last four weeks, had a clinical diagnosis of stroke, or any co-morbidities that increased the risk of stroke-like hypertension and chronic kidney disease were also excluded.

### Sample size estimation

The Kish Leslie formula was used to estimate the sample size for this cross-sectional study at a 95% confidence interval [15]. Since the estimated proportion of abnormal TCD velocities among children with SCA attending the SCD clinic of MRRH is unknown, a 50% prevalence was considered. Using a precision of 5%, a sample size of 385 was calculated.

### Study procedures

Trained research assistants with a diploma in clinical medicine collected data using a structured questionnaire. Research assistants approached children and their caretakers attending the SCA clinic, informed them about the study, screened them, and confirmed that they had HbSS by checking clinical records. After enrollment, the interview was conducted, participants were scheduled for TCD screening, and venous blood sampling.

### TCD ultrasonography

The procedure was performed by a licensed sonographer who had speciﬁc training on TCD by Cincinnati Children’s Hospital [8] with 10 years of experience doing TCD screening on patients with SCA. The procedure was done according to the STOP protocol [12]. We ensured patients lie comfortably, in a supine position, while laterally tilting the head in either position. The procedure was explained to the patients in simple language that they could understand. They were advised to be calm and stay awake, without talking or laughing during the procedure. The sonographer sat behind the head of the patient and ensured that his arms were well supported to have a firm grip on the probe. An adequate amount of the gel was applied over the temporal region, slightly above the zygomatic arch, and immediately anterior and superior in a transverse position to the tragus, and then insonation of the temporal window was done. The Middle cerebral artery (MCA), Posterior cerebral artery (PCA), Anterior cerebral artery (ACA), and the distal portion of the Distal internal carotid artery (DICA) were viewed using a 2MHz transducer to come up with the Time Average Mean of Maximum Velocity (TAMMV). The sample volume was set at 5 mm. To optimize the display, the scale and the gain were set. Each vessel was identified by its unique flow direction, depth, and Doppler signals. The sonographer adjusted the transducer position and angle to obtain the arterial velocity readings. There was an increase of the depth by 2 mm for each artery until the highest velocity was recorded, and a clear waveform was formed. Multiple measurements were done on both sides at varying depths between 40 and 70 mm to ensure accuracy. The highest TAMMV as recorded from the 4 blood vessels was used to classify risk for stroke according to the stroke STOP trial protocol as follows; normal velocities (Standard risk) < 170 cm/s; conditional velocity (Intermediate risk) 170–199 cm/s, and high risk ≥ 200 cm/s. Patients who were uncooperative or whose transtemporal acoustic windows were hard to access were not considered. This data collection procedure lasted 2 months from July to August 2024.

### Blood sample collection

Blood samples were collected by venipuncture using an ethylene diamine tetra-acetic acid (EDTA) vacutainer or purple top for CBC proﬁling and a vacutainer (red top) without anticoagulant for LDH, on the same day on which TCD was performed. An automated hemo-analyzer (Symex-xn550) was used to analyze the CBC parameters. Serum lactate dehydrogenase (LDH) was investigated using Roche Cobas Chemistry Analyzer (Roche Diagnostics Corporation, Indianapolis, IN 46250).

### Data analysis

Analyses were conducted in Stata 19 (StataCorp LLC, College Station, TX, USA).

Continuous data were summarized as means (standard deviation) or medians (interquartile range). Categorical data were summarized as frequencies (percentages).

### Predictive modelling

We used the least absolute shrinkage and selection operator (LASSO) penalized regression to select the best subset of predictors of increased TCD velocity. LASSO selects a subset of predictors by shrinking the coefficients of the least contributive variables to zero and excluding them from the model [16]. A 10-fold cross-validation was used to select the lambda, which determines the amount of coefficient shrinkage, and to generate a realistic estimation of the predictive performance of the final model, presented using the area under the curve (AUC). 10-fold cross-validation is preferable to split-sample validation because it results in better use of available data (entire dataset used in both training and validation), increased robustness (10 test sets used), reduced variance, and reduced the likelihood of obtaining overly optimistic estimates. The area under the curve estimates the ability of the model to differentiate between children with increased and normal TCD. An AUC value of 70–80% implies acceptable performance, 80–90% outstanding performance of the model to differentiate between participants with the outcome or not. The degree of agreement of the model-predicted risk of elevated TCD and observed elevated TCD among the children with SCA was evaluated using calibration belt plots. The calibration plot visually represents differences in observed frequencies and expected probabilities, as well as the localized direction of deviation at certain confidence levels. A calibration test was used to assess whether any deviations from the bisector [45° line of perfect fit] were significant.

### Model development

Based on literature from previous studies we included 16 candidate predictors in our initial model*:* age, *white blood cells, HCT, hemoglobin level, transfusion, adherence* [17] *neuropathy, Red blood cell count, heart rate* [17]*, headache, neutrophils, lymphocytes LDH* [18]*, gender, malnutrition* [17]*, admitted in last year for a complication of SCD* [19]. Neuropathy was only assessed in children aged ≥7 years. To preserve the full analytic sample (n = 385) and avoid the misleading treatment of this structured non-assessment as missing data, we created a three-category variable: neuropathy absent, neuropathy present, and not assessed due to age below 7 years. This coding was included in the LASSO model, allowing the algorithm to determine whether age-related non-assessment carried independent predictive value. After performing LASSO logistic regression with 10-fold cross-validation, 12 predictors were retained: *neuropathy, RBC, heart rate, age, adherence, headache, HCT, LDH, gender, malnutrition, transfusion, and neutrophils* in the final model using a lambda value of 0.0081707. For the 16 predictors selected for the LASSO model, there were no missing values. We have attached Table 5 as an appendix in S3 File, describing the relevance of the variables used in our model.

### Sensitivity analysis

We conducted two complementary sensitivity analyses to address the class imbalance in our outcome. First, we applied oversampling of the minority class to increase the representation of positive cases and then re-estimated model performance. Second, we examined threshold adjustment by varying the probability cut-off used to classify individuals as having elevated TCD velocity. In particular, we selected thresholds corresponding to clinically relevant trade-offs between sensitivity and specificity (e.g., 0.90 and 0.80), and evaluated the resulting impact on classification performance and model calibration.

### Ethical consideration

This study was conducted in accordance with ethical guidelines to ensure the protection and respect of participants. Approval to carry out the study was obtained from the Busitema University Research and Ethics Committee (BUREC)-BUFHS-2024–178. We obtained written informed consent from the caregivers of children who were eligible. We obtained assent from children who were at least 8 years of age.

## Results

### Participant characteristics

**Fig 1:**
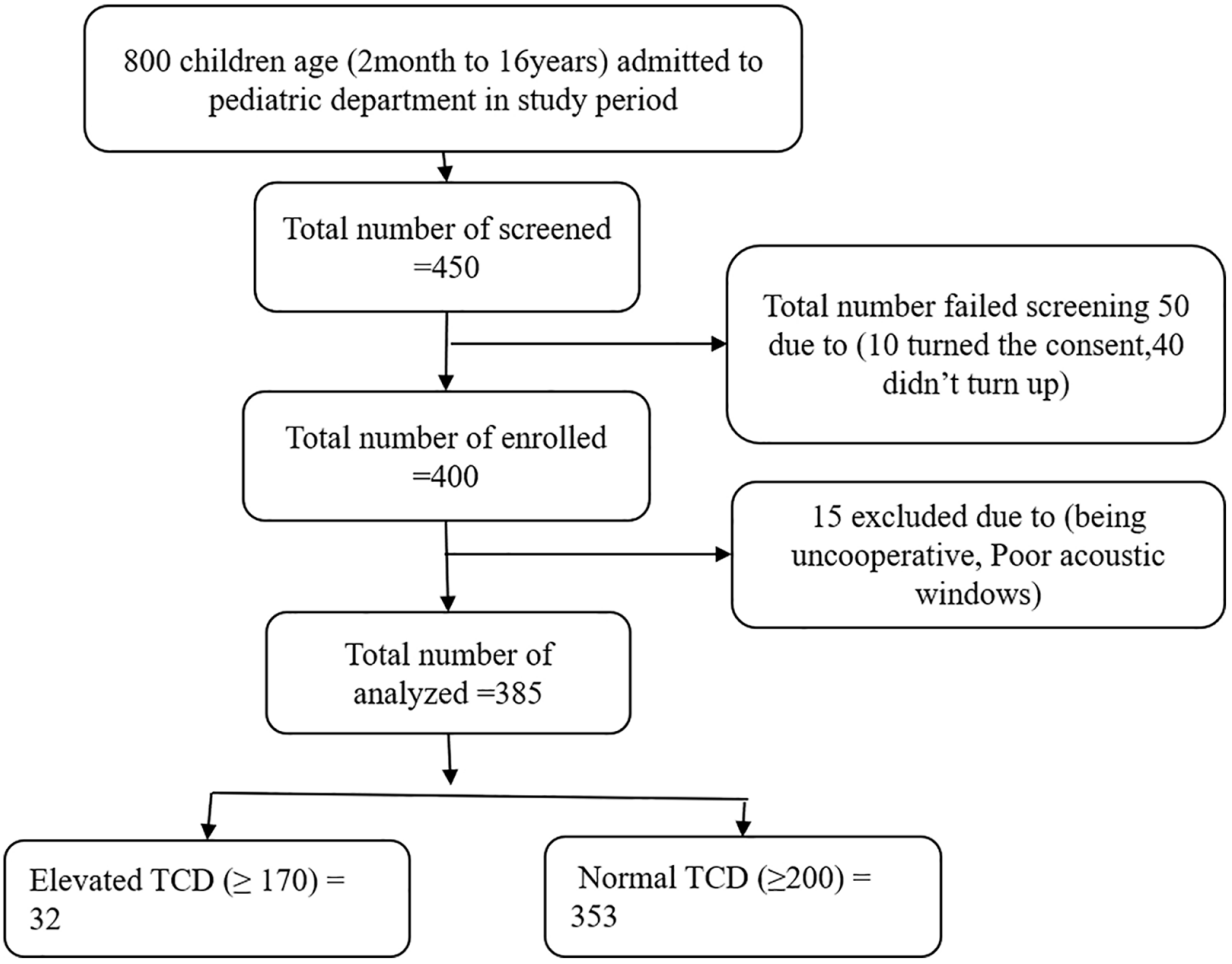
A flow chart showing screening and recruitment of children with sickle cell anemia for TCD screening.

We screened 450 children and enrolled 385, of whom 53% were females and 60.5% were aged 6–12 years. The participants had an age range of 2–16 years and a mean age of 10.3years (SD 3.8). Three-quarters (75.6% (291/385)) of the children had been initiated on hydroxyurea treatment, 40% (156/385) were fully adherent to hydroxyurea treatment. Details are in Fig 1 and Tables 1 and 2

**Table 1:** Social demographic characteristics of children with SCA attending a clinic at a regional referral hospital.

	Normal TCD	Elevated TCD	Total
	353 (91.7)	32 (8.3)	385 (100.0)
Age years **Mean (SD)**	10.44 (3.73)	8.34 (3.60)	10.27 (3.76)
Age group			
< 5	44 (12.5)	10 (31.2)	54 (14.0)
5-12	190 (53.8)	18 (56.2)	208 (54.0)
13-16	119 (33.7)	4 (12.5)	123 (31.9)
Age at first diagnosis			
1	89 (25.2)	14 (43.8)	103 (26.8)
2	52 (14.7)	3 (9.4)	55 (14.3)
3	30 (8.5)	4 (12.5)	34 (8.8)
4	33 (9.3)	1 (3.1)	34 (8.8)
≥ 5	149 (42.2)	10 (31.2)	159 (41.3)
Residence			
Rural	225 (63.9)	20 (62.5)	245 (63.8)
Urban	127 (36.1)	12 (37.5)	139 (36.2)
Gender			
Male	168 (47.6)	13 (40.6)	181 (47.0)
Female	185 (52.4)	19 (59.4)	204 (53.0)

**Table 2:** Clinical characteristics of children with SCA attending a clinic at a regional referral hospital.

	Normal TCD	Elevated TCD	Total
	353 (91.7)	32 (8.3)	385 (100.0)
Initiated on hydroxyurea			
Yes	265 (75.1)	26 (81.2)	291 (75.6)
No	88 (24.9)	6 (18.8)	94 (24.4)
Hydroxyurea Adherence			
Regularly (did not miss a day)	150 (42.5)	6 (18.8)	156 (40.5)
Missed less than 7 days a month	99 (28.0)	15 (46.9)	114 (29.6)
Missed 7 or more days in a month	18 (5.1)	4 (12.5)	22 (5.7)
Missed for a month	86 (24.4)	7 (21.9)	93 (24.2)
Heart rate^1^ (Pulse rate)			
Low	11 (3.1)	0 (0.0)	11 (2.9)
Normal	149 (42.2)	4 (12.5)	153 (39.7)
Elevated	193 (54.7)	28 (87.5)	221 (57.4)
Nutrition status			
Malnourished^2^	258 (73.1)	24 (75.0)	282 (73.2)
Normal	95 (26.9)	8 (25.0)	103 (26.8)
History of neuropathy in the past one month			
None	349 (98.9)	30 (93.8)	379 (98.4)
Yes	4 (1.1)	2 (6.2)	6 (1.6)
History of headache			
None	90(25.5)	15(46.9)	105(27.3)
Monthly	263(74.5)	17(53.1)	280(72.7)
Blood transfusion in last one year			
None	124(35.1)	7(21.9)	131(34)
Yes	229(64.9)	25(78.1)	254(66)

*Heart rate: for 2 to < 5years:normal is 92−117 bpm, for 6–12years: normal is 74−103 bpm, for 12–15 years 62−96bpm for 15−18 years 58−94bpm* [20]. ^2^ *Malnourished child is Z-score ≤ −2 SD weight for height*

### Hematological indices

The mean hemoglobin level was 7.9g/dl (1.6), most of the children had normal white blood cell count 72.2% 9278/385) and red blood cell count 61.8% (238/385). Details are in Table 3.

**Table 3:** Hematological indices of children with SCA attending a clinic at a regional referral hospital.

	Normal TCD	Elevated TCD	Total
	353 (91.7%)	32 (8.3%)	385 (100.0%)
WBC, **Mean (SD)**	14.88 (10.35)	19.24 (24.12)	15.25 (12.11)
Serum LDH, **Mean (SD)**	625.30 (253.55)	664.88 (433.47)	628.59 (272.44)
Hb **Mean (SD)**	7.96 (1.57)	7.25 (1.66)	7.90 (1.59)
WBC			
Normal (4.0–11.0)	258 (73.1)	20 (62.5)	278 (72.2)
High	95 (26.9)	12 (37.5)	107 (27.8)
RBC			
Normal (2.5–5.50)	229 (64.9)	9 (28.1)	238 (61.8)
Low	124 (35.1)	23 (71.9)	147 (38.2)
Hb			
Normal (12.00–14.00)	163 (46.2)	11 (34.4)	174 (45.2)
Low	190 (53.8)	21 (65.6)	211 (54.8)
HCT			
Normal (26.00–50.00)	79 (22.4)	2 (6.2)	81 (21.0)
Low < 26	274 (77.6)	30 (93.8)	304 (79.0)
MCV			
Normal (86–88)	152 (43.1)	19 (59.4)	171 (44.4)
Low < 86	201 (56.9)	13 (40.6)	214 (55.6)
MCH			
Normal (26–50)	277 (78.5)	29 (90.6)	306 (79.5)
Low	76 (21.5)	3 (9.4)	79 (20.5)
RDW			
Normal (11 –15)	87 (24.6)	6 (18.8)	93 (24.2)
High	266 (75.4)	26 (81.2)	292 (75.8)
Neutrophils (1.5–7.00)			
Normal	269 (76.2)	25 (78.1)	294 (76.4)
High >7	84 (23.8)	7 (21.9)	91 (23.6)
Lymphocytes			
Normal (1.00–3.70)	74 (21.0)	3 (9.4)	77 (20.0)
High>3.70	279 (79.0)	29 (90.6)	308 (80.0)
Monocytes			
Normal (0.00–0.70)	60 (17.0)	6 (18.8)	66 (17.1)
High >0.70	293 (83.0)	26 (81.2)	319 (82.9)
Basophils			
Normal (0.00–0.40)	124 (35.1)	10 (31.2)	134 (34.8)
High	229 (64.9)	22 (68.8)	251 (65.2)
Eosinophils			
Normal (0.00–0.10)	170 (48.2)	13 (40.6)	183 (47.5)
High	183 (51.8)	19 (59.4)	202 (52.5)
Serum LDH			
Normal	47 (13.3)	2 (6.2)	49 (12.7)
High	306 (86.7)	30 (93.8)	336 (87.3)

### Prevalence of elevated TCD velocity

The prevalence of elevated TCD was 8.3% (95% CI: 5.8,11.5; n = 32/385). Only 6.8% (26/385) of the children had conditional (170 to <200 cm/s) Timed Average Mean Maximum Velocity (TAMMV), and 1.6% (6/385) had high-risk (≥200 cm/s) TAMMV.

### Model predictors

**Fig 2:**
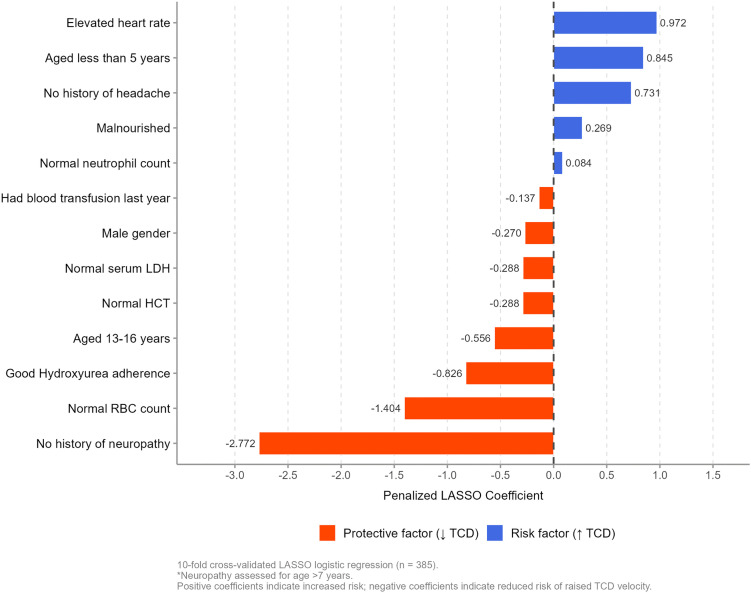
LASSO-selected predictors of raised TCD velocity among children with sickle cell anemia.

In the penalized regression model, based on a mean lambda of 0.0081707, neuropathy (β = –2.77), good hydroxyurea adherence (β = –0.83), age 13–16 years (β = –0.55), and normal RBC count (β = –1.40) predicted decreased risk of elevated TCD. In contrast, no history of headache (β = 0.73), elevated heart rate (β = 0.97), and age < 5 years (β = 0.84) predicted increased risk. The results are presented in Table 4 and visualized in Fig 2.

**Table 4:** LASSO regression for the predictors of increased TCD among children with SCA.

Predictor Variable	Penalized coefficient
Aged less than 5 years	0.8453868
Aged 13–16 years	−0.5564467
Male gender	−0.2695742
Malnourished	0.269488
No history of neuropathy	−2.771809
No history of headache	0.731085
Good hydroxyurea adherence	−0.8256155
Had blood transfusion last year	−0.1366147
Elevated heart rate	0.9722848
Normal red blood cell count	−1.404316
Normal HCT	−0.2883445
Normal neutrophil count	0.0840207
Normal serum LDH	−0.2878215

### Discrimination and distribution of risk scores

**Fig 3:**
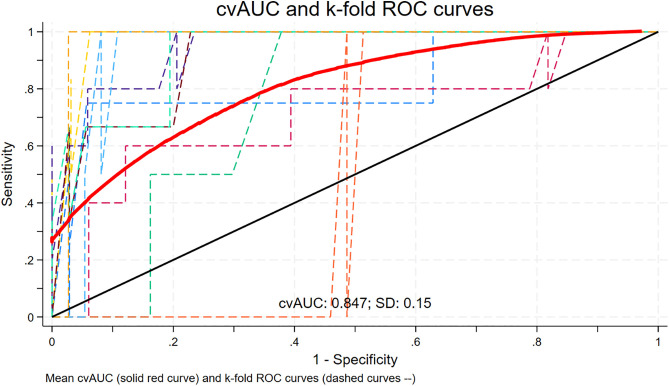
Model discrimination: ROC curve showing mean Cross-validated area under the curve (CvAUC) resulting from 10-fold cross-validation. AUC = 84.7; Bootstrap bias corrected 95% CI: 74.7, 90.8. The diagonal line represents a model that discriminates by chance (AUC = 50); the x-axis shows the proportion without elevated TCD who were incorrectly classified as having elevated TCD (false positive rate or 1 specificity); the y-axis shows the proportion with elevated TCD that were correctly classified as having elevated TCD (sensitivity or true positive rate).

Following 10-fold cross-validation to assess internal validation, the cross-validated mean AUC for the final model (Fig 3) was 84.7% (BC 95% CI: 74.7, 90.8). This meant that for a randomly selected child with SCA, there was an 84% probability that the model would correctly assign a higher risk score for a child with elevated TCD than a child with normal TCD. The model predicted risk scores for the children with SCA ranged from 0.3% to 58.8%. The median predicted risk score among the sample was 4.8% (IQR: 1.8, 11.5). Using the Liu method, the empirical optimal cutoff probability for classifying outcome was 10.2%, which yielded a sensitivity of 81% and specificity of 78%. The corresponding area under the ROC curve at this threshold was 79%, indicating good discriminatory ability. There was a higher prevalence of children with SCA with elevated TCD compared to low TCD for predicted risk scores of 10.2% and above.

### Calibration

**Fig 4:**
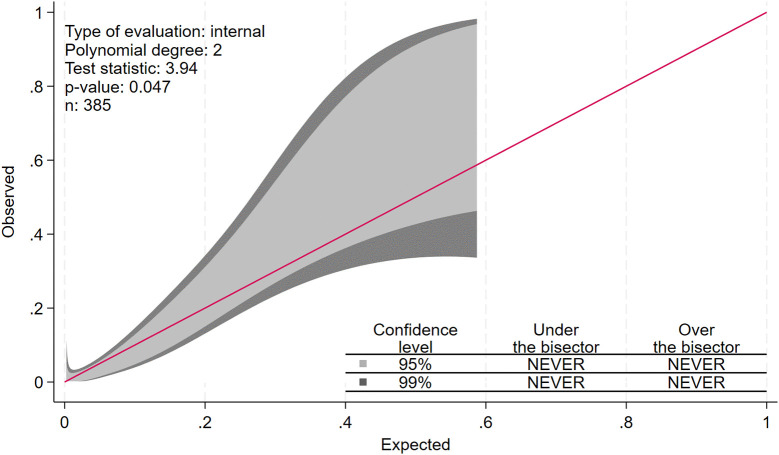
Calibration belt plot of a LASSO model to predict elevated TCD velocity among children with sickle cell anemia.

**Fig 5:**
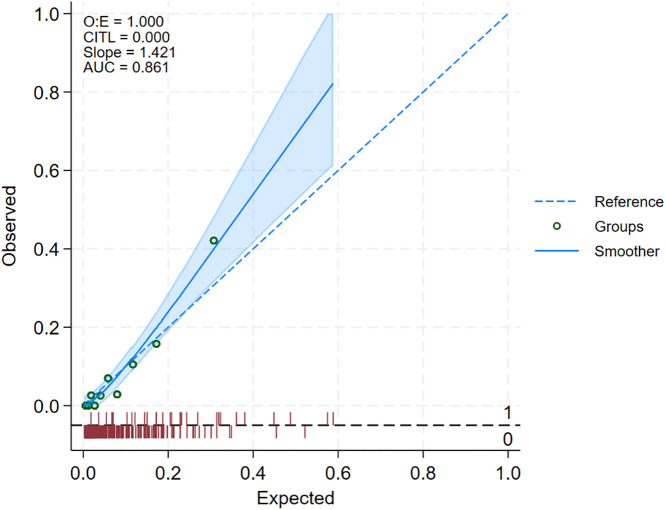
Calibration slope plot of a LASSO model to predict elevated TCD velocity among children with sickle cell anemia.

Calibration of the prediction model was assessed using the calibration belt approach. The calibration curve closely followed the bisector line, and the 95% and 99% confidence bands did not cross the line of perfect calibration across the range of predicted probabilities, indicating no systematic over- or under-prediction. The calibration test yielded a test statistic of 3.94 (p = 0.047) based on a second-order polynomial with n = 385, suggesting borderline statistical evidence of deviation from perfect calibration. However, this deviation was not directional, as the model predictions were never significantly above or below the bisector at either the 95% or 99% confidence level. This is shown in Figs 4 and 5.

## Discussion

### Prevalence of elevated TCD

The prevalence of elevated TCD velocity among children with sickle cell anemia was 8.3% in this study. This was quite low and may be explained by the fact that more than 75% of the children had been initiated on routine hydroxyurea at the sickle cell clinic. At the clinic, children are initiated on hydroxyurea at dose ranges of 15–20 mg/kg.

This prevalence is comparable to the NOHARM study in Uganda, which found a prevalence of 11% after 2 years of follow-up [21]. This can be explained by the fact that children in the NOHARM trial received low-dose hydroxyurea. Our prevalence is also similar to the DISPLACE multi-center cohorts in the United States (8.3%) [22]. This similarity may be explained by the shared pathophysiology of cerebral vasculopathy in SCA and similar age distributions of study populations in the cohort [22]. The DISPLACE study used similar predictors (socio-demographic, anthropometry, and hematologic parameters) for the LASSO regression, the study also enrolled children within a similar age range (2–16 years). These factors suggest that the underlying risk of elevated TCD in children with SCA may be relatively stable across diverse settings.

However, clinical trials that implemented escalation of hydroxyurea to the maximum tolerated dose reported a much lower prevalence in comparison to our findings. These include the REACH trial, which had a 3.4% prevalence [23], and another study in Uganda 5.7% [24]. Studies done among children with the entire cohort not initiated on hydroxyurea have reported a prevalence of elevated TCD of more than 30% [21,25]. This highlights the key role of hydroxyurea as a disease-modifying drug in the management of sickle cell anemia.

### Predictors of elevated TCD

Overall, the model identified history of neuropathy, red blood cell count, hydroxyurea adherence, age, and heart rate as the most relevant factors to predict risk of elevated TCD among children with SCA. Our model gave us an AUC above 80%, which shows that the true discriminative ability of the model is acceptable and has a good overall performance.

The 12 predictors retained after LASSO regression (*neuropathy, RBC, heart rate, age, adherence, headache, HCT, LDH, gender, malnutrition, transfusion, neutrophils)* have also been associated with risk of elevated TCD among children with SCA in previous studies that have used routine regression models [26–28]. The coefficients from the LASSO model are measures of prediction and not causation, therefore the results may not be fully explained by biological plausibility.

The results show that identifying children at risk of elevated TCD velocity is possible using a combination of socio-demographic characteristics (age, gender), clinical history and examination (history of headache, neuropathy, adherence to hydroxyurea, blood transfusion within 1 year, and nutrition status), and routine laboratory investigations (RBC, HCT, neutrophils, serum LDH). Our analysis offers a plausible screening tool to determine individualized risk of elevated TCD among children with SCA. This will guide patient management and utilization of limited resources, such as hydroxyurea and blood, by those considered to be at higher risk. Clinicians can use the model to stratify patients at higher risk of elevated TCD velocity, and intervene accordingly without doing a TCD scan which is usually unavailable.

System constraints, such as having few ultrasound machines and limited personnel to do routine TCD screening, make relying on self-reported measures and routine CBC tests inevitable alternatives. Ably identifying children with elevated TCD and at risk of stroke will aid timely intervention and ensure better quality of life for children with SCA. Individualized risk assessment approaches have the potential to advance our understanding, specifically in identifying children at risk for poor health outcomes even before they occur.

Patient history and laboratory investigations are routinely taken and documented, but are underutilized to inform clinical decision-making.

### Strengths and limitations

Our study generates data to inform the identification of clinically relevant factors that can guide individualized approaches to screening of children with SCA at risk of having elevated TCD. Prior studies have used regression-based analyses to measure the association between elevated TCD and different characteristics; these are necessary in understanding causation.

From these studies, numerous factors have been associated with elevated TCD. There is a need to identify potential clinically relevant predictors (including their relative predictive value) to inform individualized assessments for the risk of elevated TCD. These findings should be interpreted in consideration of the study limitations. The model achieves strong discrimination (AUC = 0.861); however, the calibration slope >1 (1.421) suggests that the predicted risks are extreme, whereby lower-risk patients are overestimated, and higher-risk patients are underestimated. This could be due to the imbalanced distribution of the outcome. Our study had only 32 outcomes, and our final model retained 12 predictors. We had a very low events per parameter ratio, and our LASSO model may have overfitted the AUC estimate.

Adherence was assessed from patient history, and we cannot rule out bias due to social desirability. Although the AUC of 87.4% may be acceptable for the development of an initial screening tool, there is a need for further expansion and validation work to optimize its accuracy before deployment in a clinical setting to guide clinical decisions. This was a single-site study; therefore, we cannot confirm the external validity of our results, and our findings may not apply to children in other settings. Our findings may also not be generalizable to other low-resource settings where hydroxyurea use is lower or completely absent.

The clinical utility of the score, as well as the feasibility and acceptability of this tool, also need to be measured before being used in practice. Although a factor may be statistically significantly associated with poor adherence, as seen in previous research, it may not necessarily have predictive power, and not contribute to predicting elevated TCD, as was indicated by the subset of predictors excluded after lasso regression. Although *white blood cells, lymphocytes, hemoglobin level, admitted in the last year,* were not retained as predictors in the final model, future studies with more robust designs should also consider exploring these interactions further, due to their biological plausibility and association with elevated TCD velocity.

## Conclusions

Our results support the use of prediction modelling techniques to develop screening tools to identify children with elevated TCD. Using routinely collected data from patient history, physical examination, and laboratory investigations, a risk score can be developed for use among children with SCA. However, further exploration of these factors using more robust study designs and external validation is required before implementation.

## Supporting information

S1 File STROBE Statement checklist. Checklist of items that should be included in reports of observational studies.
(DOCX)

S2 File Data set for children with SCD attending a clinic in Eastern Uganda. (XLS)

S3 File Appendix: Table 5. A description of different variables that can be used to predict raised TCD among children with SCD [17–19]. (DOCX)
