# Supplementary material for: Predicting elevated transcranial doppler velocity among patients with sickle cell anemia in Uganda: A cross-sectional study
Source: PLoS One. 2026 Jun 23;21(6):e0351700. doi: 10.1371/journal.pone.0351700 (PMC13289871; doi:10.1371/journal.pone.0351700)
Supplement: S1 File — Checklist of items that should be included in reports of observational studies. (DOCX) [file pone.0351700.s001.docx]

STROBE Statement—checklist of items that should be included in reports of observational studies

|  | Item No. | Recommendation | Page  No. | Relevant text from manuscript |
| --- | --- | --- | --- | --- |
| **Title and abstract** | 1 | (*a*) Indicate the study’s design with a commonly used term in the title or the abstract | 1 | Predicting elevated transcranial doppler velocity among patients with sickle cell anemia in Uganda: a cross-sectional study |
|  |  | (*b*) Provide in the abstract an informative and balanced summary of what was done and what was found | 2,3 | We conducted a cross sectional study from 1^st^ July 2024 to 30^th^ August 2024 among children with SCA attending the Sickle Cell Clinic. We developed a risk-prediction model for elevated TCD (> 170 cm/s) using sociodemographic, hematological, and clinical factors. We used the least absolute shrinkage and selection operator (LASSO) penalized regression to select the best subset of predictors of increased TCD velocity. |
| Introduction | | | |  |
| Background/rationale | 2 | Explain the scientific background and rationale for the investigation being reported | 3, 4 | Transcranial doppler velocity predicts risk of stroke among children with sickle cell disease. Previous studies in Uganda have documented a high prevalence of abnormal TCD among children with SCA. Unfortunately, TCD screening is not routinely done in developing countries due to limited ultrasound machines, as well as few qualified practitioners to perform TCD screening. There is need to generate data to guide accurate prediction of elevated TCD among children with SCA. This will guide screening for children at risk of developing stroke. |
| Objectives | 3 | State specific objectives, including any prespecified hypotheses | 4 | Using data collected from a cross-sectional study among children with sickle cell, we utilized prediction modelling techniques to: (1) identify a model comprising a combination of a subset of multi-level factors that best predicts individualized risk of elevated TCD velocity among children with SCA. (2) Evaluate the extent to which the derived model can discriminate between normal and elevated TCD velocity among children with SCA. (3) Assess the agreement between model predicted and actual elevated TCD velocities among children with SCA (calibration). |
| Methods | | | |  |
| Study design | 4 | Present key elements of study design early in the paper | 4 | We conducted a cross sectional study from 1^st^ July 2024 to 30^th^ August |
| Setting | 5 | Describe the setting, locations, and relevant dates, including periods of recruitment, exposure, follow-up, and data collection | 4,5, 6 | We conducted a cross sectional study from 1^st^ July 2024 to 30^th^ August 2024 among children with SCA attending the Sickle Cell Clinic at Mbale Regional Referral Hospital (MRRH). |
| Participants | 6 | (*a*) *Cohort study*—Give the eligibility criteria, and the sources and methods of selection of participants. Describe methods of follow-up  *Case-control study*—Give the eligibility criteria, and the sources and methods of case ascertainment and control selection. Give the rationale for the choice of cases and controls  *Cross-sectional study*—Give the eligibility criteria, and the sources and methods of selection of participants | 4 | We included children aged 2 – 16 years with SCA attending the Sickle Cell Clinic at MRRH and were clinically stable. We excluded patients with current presentation of an acute illness such as pain crisis, fever, acute chest syndrome or other SCA-related acute complications, these were admitted and given appropriate treatment. Patients who had received a blood transfusion in the last four weeks, had a clinical diagnosis of stroke or any co-morbidities that increased the risk of stroke-like hypertension and chronic kidney disease were also excluded. |
|  |  | (*b*) *Cohort study*—For matched studies, give matching criteria and number of exposed and unexposed  *Case-control study*—For matched studies, give matching criteria and the number of controls per case |  |  |
| Variables | 7 | Clearly define all outcomes, exposures, predictors, potential confounders, and effect modifiers. Give diagnostic criteria, if applicable | 7 | The outcome variable was elevated TCD > 170cm/s. The different exposure variables included: sociodemographic characteristics, use of hydroxyurea and laboratory indices. |
| Data sources/ measurement | 8* | For each variable of interest, give sources of data and details of methods of assessment (measurement). Describe comparability of assessment methods if there is more than one group | *5,6* | Trained research assistants having a diploma in clinical medicine collected data using a structured questionnaire. Research assistants, approached children and their care takers attending the SCA clinic, informed them about the study, screened them, confirmed that they had HbSS by checking clinical records. After enrollment, the interview was conducted, participants were scheduled for TCD screening, and venous blood sampling. |
| Bias | 9 | Describe any efforts to address potential sources of bias | N/A |  |
| Study size | 10 | Explain how the study size was arrived at | 5 | The Kish Leslie formula was used to estimate the sample size for this cross-sectional study at a 95% confidence interval (13). The estimated proportion of abnormal TCD velocities among children with SCA attending the SCD clinic of MRRH is unknown, a 50% prevalence was considered and a sample size of 385 was calculated. |

Continued on next page

| Quantitative variables | 11 | Explain how quantitative variables were handled in the analyses. If applicable, describe which groupings were chosen and why | 6 | Analyses were conducted in Stata 19 (StataCorp LLC, College Station, TX, USA).  Continuous data were summarized as means (standard deviation) or medians (interquartile range). Categorical data were summarized as frequencies (percentages). |
| --- | --- | --- | --- | --- |
| Statistical methods | 12 | (*a*) Describe all statistical methods, including those used to control for confounding | 7 | We used the least absolute shrinkage and selection operator (LASSO) penalized regression to select the best subset of predictors of increased TCD velocity. Model performance was assessed by determining the discrimination using the area under the curve and calibration by drawing a calibration plot. |
|  |  | (*b*) Describe any methods used to examine subgroups and interactions |  |  |
|  |  | (*c*) Explain how missing data were addressed |  |  |
|  |  | (*d*) *Cohort study*—If applicable, explain how loss to follow-up was addressed  *Case-control study*—If applicable, explain how matching of cases and controls was addressed  *Cross-sectional study*—If applicable, describe analytical methods taking account of sampling strategy |  |  |
|  |  | (*e*) Describe any sensitivity analyses | 8 | We conducted two complementary sensitivity analyses to address the class imbalance in our outcome. First, we applied oversampling of the minority class to increase the representation of positive cases and then re-estimated model performance. Second, we examined threshold adjustment by varying the probability cut-off used to classify individuals as high risk. In particular, we selected thresholds corresponding to clinically relevant trade-offs between sensitivity and specificity (e.g., 0.90 and 0.80), and evaluated the resulting impact on classification performance and model calibration. |
| Results | | | | |
| Participants | 13* | (a) Report numbers of individuals at each stage of study—eg numbers potentially eligible, examined for eligibility, confirmed eligible, included in the study, completing follow-up, and analysed | 8 | We screened 450 children and enrolled 385. Reasons for exclusion are detailed in figure 1. |
|  |  | (b) Give reasons for non-participation at each stage |  |  |
|  |  | (c) Consider use of a flow diagram | 9 |  |
| Descriptive data | 14* | (a) Give characteristics of study participants (eg demographic, clinical, social) and information on exposures and potential confounders | 8 | We screened 450 children and enrolled 385, of whom 53% were females and 60.5% were aged 6 to 12 years. The participants had an age range of 2 to 16 years and a mean age of 10.3years (SD 3.8). Three-quarters (75.6% (291/385)) of the children had been initiated on hydroxyurea treatment, 40% (156/385) were fully adherent to hydroxyurea treatment. Details are in Figure 1, Table 1 and Table 2 |
|  |  | (b) Indicate number of participants with missing data for each variable of interest |  |  |
|  |  | (c) *Cohort study*—Summarise follow-up time (eg, average and total amount) |  |  |
| Outcome data | 15* | *Cohort study*—Report numbers of outcome events or summary measures over time |  |  |
|  |  | *Case-control study—*Report numbers in each exposure category, or summary measures of exposure |  |  |
|  |  | *Cross-sectional study—*Report numbers of outcome events or summary measures | *13* |  |
| Main results | 16 | (*a*) Give unadjusted estimates and, if applicable, confounder-adjusted estimates and their precision (eg, 95% confidence interval). Make clear which confounders were adjusted for and why they were included) | 7,8 | Based on literature from previous studies we included 16 candidate predictors in our initial model*:* *neuropathy, RBC, heart rate, age, adherence, headache, HCT, LDH, gender, malnutrition, transfusion, neutrophils, white blood cells, lymphocytes, hemoglobin level, admitted in last year.* After performing LASSO logistic regression with 10-fold cross-validation, 12 predictors were retained: *neuropathy, RBC, heart rate, age, adherence, headache, HCT, LDH, gender, malnutrition, transfusion, neutrophils* in the final model using a lambda value of 0.0081707. |
|  |  | (*b*) Report category boundaries when continuous variables were categorized | 9, 10,11 | Table 1, Table 2, Table 3 |
|  |  | (*c*) If relevant, consider translating estimates of relative risk into absolute risk for a meaningful time period |  |  |

Continued on next page

| Other analyses | 17 | Report other analyses done—eg analyses of subgroups and interactions, and sensitivity analyses | 8 | We conducted two complementary sensitivity analyses to address the class imbalance in our outcome. First, we applied oversampling of the minority class to increase the representation of positive cases and then re-estimated model performance. Second, we examined threshold adjustment by varying the probability cut-off used to classify individuals as high risk. In particular, we selected thresholds corresponding to clinically relevant trade-offs between sensitivity and specificity (e.g., 0.90 and 0.80), and evaluated the resulting impact on classification performance and model calibration. |
| --- | --- | --- | --- | --- |
| Discussion | | | | |
| Key results | 18 | Summarise key results with reference to study objectives | 15. 16 | The prevalence of elevated transcranial doppler velocity among children with sickle cell anemia was 8.3% in this study.  Overall, the model identified history of neuropathy, red blood cell count, hydroxyurea adherence, age, and heart rate as the most relevant factors to predict risk of elevated TCD among children SCA. Our model gave us an AUC above 80%, this shows that the true discriminative ability of the model is acceptable and has a good overall performance. |
| Limitations | 19 | Discuss limitations of the study, taking into account sources of potential bias or imprecision. Discuss both direction and magnitude of any potential bias | 17 | Our study generates data to inform identification of clinically relevant factors that can guide individualized approaches to screening of children with SCA at risk of having elevated TCD.  Although the AUC of 87.4% may be acceptable for the development of an initial screening tool, there is a need for further expansion and validation work to optimize its accuracy before deployment in a clinical setting to guide clinical decisions. |
| Interpretation | 20 | Give a cautious overall interpretation of results considering objectives, limitations, multiplicity of analyses, results from similar studies, and other relevant evidence | 15, 16, 17 |  |
| Generalisability | 21 | Discuss the generalisability (external validity) of the study results | 18 | This was a single site study, so our findings may not be generalizable to the general population. |
| Other information | |  | | |
| Funding | 22 | Give the source of funding and the role of the funders for the present study and, if applicable, for the original study on which the present article is based | 18, 19 | This research study was funded by “National Institutes of Health’s,  FOGARTY INTERNATIONAL CENTER to “Enhancing Research Capacity for Sickle Cell  Disease and related NCDs across the Lifespan in Uganda, Enrich Project under award number  D43TW012466. The funders had no role in study design, implementation and data analysis. |

*Give information separately for cases and controls in case-control studies and, if applicable, for exposed and unexposed groups in cohort and cross-sectional studies.

**Note:** An Explanation and Elaboration article discusses each checklist item and gives methodological background and published examples of transparent reporting. The STROBE checklist is best used in conjunction with this article (freely available on the Web sites of PLoS Medicine at http://www.plosmedicine.org/, Annals of Internal Medicine at http://www.annals.org/, and Epidemiology at http://www.epidem.com/). Information on the STROBE Initiative is available at [www.strobe-statement.org](http://www.strobe-statement.org).
