# Supplementary material for: Predicting elevated transcranial doppler velocity among patients with sickle cell anemia in Uganda: A cross-sectional study
Source: PLoS One. 2026 Jun 23;21(6):e0351700. doi: 10.1371/journal.pone.0351700 (PMC13289871; doi:10.1371/journal.pone.0351700)
Supplement: S3 File — (DOCX) [file pone.0351700.s003.docx]

**Appendix: Table 5. A description of different variables that can be used to predict raised TCD among children with SCD (17-19)**

| Predictor | Rationale |
| --- | --- |
| Age | Younger children have higher cerebral blood velocities; age is a recognized risk modifier for stroke in SCD |
| Heart rate | Elevated heart rate reflects compensatory hemodynamics due to anemia, potentially increasing cerebral blood flow velocity |
| Red blood cell count | Low red blood cell count indicates anemia severity; anemia drives increased cerebral flow, elevating TCD |
| Hemoglobin level | Low hemoglobin correlates with higher TCD velocities; a key marker of anemia and elevated TCD |
| Hematocrit | Low hematocrit increases cerebral flow and stroke risk. |
| Mean corpuscular volume | Reflects red cell size; abnormalities can indicate hemolysis and influence cerebral hemodynamics |
| Mean corpuscular hemoglobin | Related to oxygen-carrying capacity; may influence cerebral flow |
| White blood cell count | Elevated white blood cell count indicates systemic inflammation, and is associated with vasculopathy and raised TCD |
| Neutrophils | This is a marker of inflammation; high neutrophil counts are linked to increased TCD |
| Lymphocytes | Lymphocytes are also a marker of inflammation, and associated with elevated TCD |
| Lactate dehydrogenase | Lactate dehydrogenase is a marker of hemolysis; higher LDH is associated with vascular complications and elevated TCD |
| Adherence to hydroxyurea | Hydroxyurea reduces hemolysis and TCD velocity; adherence predicts cerebrovascular protection |
| Headache/ neuropathy | These are clinical indicators of cerebral ischemia and are associated with cerebrovascular events |
| Nutrition status | Poor nutritional status can exacerbate anemia and affect cerebrovascular health |
| Number of blood transfusions | Blood transfusions reduce anemia severity and TCD |
| Admission for complication of SCD | Acute or chronic complications increase hemolysis, inflammation, and cerebral hemodynamic stress |
